# Costs incurred by patients with oral potentially malignant disorders: is there a public health need for financial protection in India?

**DOI:** 10.1186/s13104-021-05814-2

**Published:** 2021-10-24

**Authors:** Jay R. Patel, Mihir P. Rupani

**Affiliations:** 1 Department of Community Medicine, Government Medical College Bhavnagar (Maharaja Krishnakumarsinhji Bhavnagar University), Near ST bus stand, Jail Road, Bhavnagar, Gujarat 364001 India; 2 Division of Clinical Epidemiology, ICMR-National Institute of Occupational Health (NIOH), Meghani Nagar, Ahmedabad, Gujarat 380016 India

**Keywords:** Oral precancerous lesions, Catastrophic costs, Oral cancer, Cash transfer schemes, Financial protection, Tobacco cessation

## Abstract

**Objectives:**

Financial protection mechanisms are in place to overcome the costs of a few diseases in India. Our objective was to estimate the costs incurred due to Oral Potentially Malignant Disorders (OPMD) and to determine predictors of such costs.

**Results:**

We found that the median (Interquartile range IQR) total costs of OPMD was Indian Rupees (INR) 500 (350–750), direct medical costs was INR 0 (0–50), direct non-medical costs was INR 150 (40–200) and indirect costs was INR 350 (250–500). The travel cost to attend the health facilities [INR 100 (40–150)] and the patient’s loss of wages [INR 200 (150–400)] mainly accounted for the direct non-medical and indirect costs respectively. The median expenditure on smokeless and smoking forms of tobacco was INR 6000 (5400–7200) and INR 2400 (1800–3600) respectively. On multiple linear regression analysis, rural residence, belonging to below poverty line family, being a sole earner in the family, number of months since diagnosis and first visit at a private provider were found to be the significant predictors of total costs of OPMD. Financial protection mechanisms are needed for covering the direct non-medical and indirect costs. Early management of OPMD might mitigate the costs of OPMD.

**Supplementary Information:**

The online version contains supplementary material available at 10.1186/s13104-021-05814-2.

## Introduction

Oral cancer is almost always preceded by clinically visible lesions that are noncancerous, to begin with, and which have therefore been termed oral potentially malignant disorders (OPMD) [1, 2]. Oral submucous fibrosis (OSMF), leukoplakia, erythroplakia, and lichen planus are the most commonly described OPMD [1, 2]. Globally, the burden of OPMD was reported to be 4.5%, majorly contributed by OSMF (5%) and leukoplakia (4%) [3]. The highest prevalence rates were among the Asian (11%) and South American/Caribbean populations (4%), while the lowest was observed in North America (0.11%) [3]. In India, a varying prevalence has been reported (4–58%) [4–7], 10% being the overall prevalence in western India [5].

Existing literature on costs of OPMD, none from India, focused on the costs borne by the healthcare facilities in treating these patients [8, 9]. The average annual cost borne by a hospital for a patient with oral lichen planus was reported as £400 (~ 36 000 Indian Rupees—INR) and the cost of laser surgery for OPMD was reported as £998 (~ INR 89 000) during 2015–16 [8, 9]. Evidence is scarce on costs incurred by patients with OPMD and on the predictors associated with such costs in India. Evidence suggests that financial protection mechanisms are beneficial in reducing morbidity as well as mortality among cancer patients [10, 11]. We conducted this study to estimate the costs incurred by patients for the care of OPMD and to determine the predictors of such costs in the Bhavnagar district of Gujarat (western India).

## Main text

### Methods

#### Study design, setting and participants

We conducted a cross-sectional study in the outpatient department (OPD) of the Otorhinolaryngology Department of Sir Takhtsinhji Hospital of Bhavnagar district of Gujarat from July–September 2019. The population of Bhavnagar district and Bhavnagar city as per census 2011 is around 2.8 million and 0.6 million respectively (a sparsely-populated semi-urban and rural setting) [12]. Patients ≥ 18 years and diagnosed with OPMD by an Otorhinolaryngology specialist at our hospital were included in the study.

#### Sample size

A pilot study was conducted among 40 OPMD patients in the hospital, which reported the standard deviation of total costs of OPMD as 1133. A sample size of 219 was calculated using the formula N = 4Z_α_^2^S^2^/W^2^,[13] considering 95% confidence level (Z_α_ = 1.96), the desired width of the confidence interval (W) as 300, and a standard deviation (S) of 1133.

#### Recruitment and sampling

The principal investigator (PI—first author) enrolled patients in the study through the register maintained by the staff nurse of Otorhinolaryngology OPD. On an average day in the Otorhinolaryngology OPD of our hospital manages approximately 10–15 patients with OPMD. Every second patient was enrolled consecutively in the study. After the written informed consent procedure, the questionnaire was administered by the PI.

#### Study tool

The questionnaire used for the study was a validated tool of the World Health Organization (WHO) for estimating direct medical, direct non-medical and indirect costs among tuberculosis patients, which was adapted for estimating costs among OPMD patients [14].

#### Cost survey

The costs incurred by patients were summed up for the visits they made starting from the symptoms of OPMD till they were diagnosed or treated at our hospital (that is, till the time of interview). The costs were categorized as direct medical costs (costs for consultation, hospital admission, medicine, radiology, laboratory, and any diagnostic procedures), direct non-medical costs (costs for travel, food, and accommodation) and indirect costs. Indirect costs included patient’s and accompanying member’s loss of wages, and monthly family income before symptoms of OPMD minus monthly family income at the time of interview. The total costs included the sum of direct medical, direct non-medical, and indirect costs incurred by the patients for the care of OPMD. The expenditure on consuming tobacco (both smokeless as well as smoking forms) was calculated from the time they first started their consumption until the time of the interview (it was not added to any category of costs of OPMD). During the study period, on an average, one US$ equalled 70 INR.

#### Variables

The primary outcome variable was the total healthcare costs incurred by OPMD patients as a continuous variable. The predictor variables were age in years, male gender, residence (rural vs. urban), years of education, occupation requiring labour, being a sole earner in the family, belonging to below poverty line family, having OSMF, time since diagnosis, time since consuming smokeless tobacco and having the first visit at a private provider.

#### Statistical analysis

The healthcare costs (being a continuous variable) was expressed in terms of median and interquartile range (IQR) as the skewness (4.7) and kurtosis (33.3) values suggested a lack of normality. Uni-variable linear regression analysis was performed to find out predictors to be included in the multi-variable analysis. The predictor variables with p-value < 0.2 on uni-variable regression were included in the multivariable analysis. Multiple linear regression analysis was applied using the “Enter” method for finding out the significant predictors of total healthcare costs of OPMD. The difference was said to be significant when the p-value was < 0.05.

### Results

#### Characteristics of patients

A total of 223 eligible patients were enrolled in the study (4 patients did not agree to participate in the study). The median age of the patients was 35 (IQR 28–45) years, 78% were male, 24% had no schooling, 75% belonged to other backward classes (OBC), 47% were living in the rural area and 71% had an occupation requiring labour (Table 1). The median family income was Indian Rupees (INR) 15 000 (IQR 10 000–20 000), 41% were belonging to below poverty line family and 27% were sole earners in their family. The median expenditure of consuming smokeless and smoking forms of tobacco was INR 6000 (IQR 5400–7200) and INR 2400 (IQR 1800–3600) respectively. Seventy-five percent of patients were newly diagnosed, 28% were on treatment for OPMD and 8% had first visited a private provider.

**Table 1:** Characteristic of patients with oral potentially malignant disorder at Bhavnagar during July–September 2019 (n = 219) [one US$ = 70 INR]

Characteristic	Number (%) or median (IQR)
Socio-demographic characteristic	
Age in years	35 (28–45)
Male	170 (78)
Years of education	10 (8–12)
Educational status	
No schooling	52 (24)
Had schooling	167 (76)
Married	174 (79)
Caste	
Other backward classes (OBC)	164 (75)
Scheduled caste (SC) / Scheduled tribe (ST)	19 (9)
General	36 (16)
Nuclear family	108 (49)
Rural residence	103 (47)
Occupation requiring labour	155 (71)
Economic characteristic	
Family income in Indian Rupees (INR) per month	15 000 (10 000–20 000)
Belonging to below poverty line family	89 (41)
Sole earner in the family	59 (27)
Tobacco consumption	
Type of tobacco	
Smokeless tobacco (SLT)	193 (88)
Smoking	5 (2)
Both type	21 (10)
Age of starting SLT consumption in years	25 (20–26)
Number of years of SLT consumption	10 (6–16)
Number of packets of SLT per day	3 (3–5)
Expenditure on SLT in INR	6000 (5400–7200)
Age of starting tobacco smoking in years	30 (24–40)
Number of years of tobacco smoking	5 (4–25)
Number of *“bidi”* smoked per day	5 (5–14)
Expenditure on tobacco smoking in INR	2400 (1800–3600)
Type of OPMD	
Oral submucous fibrosis (OSMF)	168 (77)
Leukoplakia	32 (15)
Erythroplakia	15 (7)
Lichen planus	4 (2)
Clinical characteristics	
Newly diagnosed	164 (75)
On treatment	61 (28)
Number of months since diagnosis	0 (0–1)
Number of months on treatment	0 (0–0)
First visit at a private provider	18 (8)

*OPMD* Oral Potentially malignant disorders, *INR* Indian Rupees, *IQR* Inter−quartile range

#### Cost burden of OPMD

The direct medical costs incurred by patients with OPMD were low and were contributed mainly by costs for the purchase of medicines [median INR 0 (IQR 0–50)] (Table 2). The median of the direct non-medical costs was INR 150 (IQR 40–200). The travel cost to attend the health facilities [median INR 100 (IQR 40–150)] mainly accounted for the direct non-medical costs. The median indirect costs among the patients were INR 350 (IQR 250–500). The indirect costs were mainly contributed by the patient’s loss of wages [median INR 200 (IQR 150–400)]. The median total costs of OPMD was INR 500 (IQR 350–750) and was mainly contributed by the indirect costs.

**Table 2:** Median (IQR) in Indian Rupee (INR) of costs incurred by patients with oral potentially malignant disorders at Bhavnagar during July–September 2019 (n = 219) [one US$ = 70 INR]

Costs of OPMD	Median (IQR)
Total costs	500 (350–750)
Direct medical cost	0 (0–50)
Consultation charges	0 (0–0)
Hospital admission charges	0 (0–0)
Medicine cost	0 (0–50)
Radiology cost	0 (0–0)
Laboratory cost	0 (0–0)
Procedure cost	0 (0–0)
Direct non-medical cost	150 (40–200)
Travel cost	100 (40–150)
Food cost	50 (0–100)
Accommodation cost	0 (0–0)
Indirect costs	350 (250–500)
Patient's loss of wages	200 (150–400)
Accompanying member's loss of wages	0 (0–200)

*IQR* inter−quartile range, *OPMD* Oral potentially malignant disorders

#### Predictors of costs of OPMD

Age in years, male gender, rural residence, being a sole earner in the family, belonging to below poverty line family, number of months since diagnosis, number of years since consuming smokeless tobacco, and having the first visit at a private provider were the variables found to have a p-value < 0.2 on unadjusted uni-variable linear regression analysis [see Additional file 1].

On performing multiple linear regression, rural residence, belonging to below poverty line family, being a sole earner in the family, number of months since diagnosis, and first visit at a private provider were found to be significantly predicting total costs of OPMD (Table 3). Rural residence, increase in one month since diagnosis and having the first visit at a private provider increased the total costs of OPMD by INR 1.2, 1.5, and 1.7 respectively. Belonging to below the poverty line family and being a sole earner in family decreased the total costs of OPMD by INR 1.2 and 1.3 respectively. The results of the regression indicated that the predictors explained 45.6% of the variance [adjusted R^2^ = 0.456, F(8,205) = 23, p < 0.001]. Age in years, male gender, and the number of years since consuming smokeless tobacco were not significantly predicting costs of OPMD.

**Table 3:** Multiple linear regression analysis for variables predicting total costs of OPMD at Bhavnagar during July–September 2019 (n = 219)

Variables	Beta-coefficients	95% C.I. of beta-coefficients	P-value
Constant	503.9	318.2–797.8	< 0.001
Age in years	1.006	− 1.005 to 1.017	0.317
Male gender	− 1.037	− 1.226 to 1.139	0.665
Rural residence	1.176	1.029–1.345	0.017
Belonging to below poverty line family	− 1.18	− 1.347 to − 1.035	0.014
Sole earner in family	− 1.264	− 1.468 to − 1.088	0.002
Number of months since diagnosis	1.477	1.353 – 1.612	< 0.001
Number of years since consuming smokeless tobacco	1.004	− 1.012 to 1.021	0.591
First visit at a private provider	1.719	1.348–2.193	< 0.001

*OPMD* Oral potentially malignant disorders

### Discussion

Patients with OPMD incurred lower direct medical and total healthcare costs when compared with their monthly family incomes in this study setting. As might be the case with patients managed at a government hospital, patients with OPMD in the present study incurred lower direct medical costs due to free diagnostic and treatment services. Over 90% of patients first visited our facility (government-run) for the management of OPMD. The low percentage of patients visiting private providers indicates their healthcare-seeking behaviour towards the public health system. Almost half of the patients resided in rural areas where specialist services of the private sector are scarce. Also, since three-fourth of the patients were diagnosed on the first visit when the cost survey took place, the costs for pre-diagnostic visits elsewhere were not incurred. None of the patients required hospitalization and were managed on an OPD basis, cancelling out costs of hospitalization.

The loss of wages of patients with OPMD and the travel cost for attending health facilities were the prominent costs reported in this study. Early identification and appropriate case management of OPMD would be pertinent in reducing the costs. The current study found substantially high expenditure on tobacco consumption (especially for SLT) among OPMD patients. Studies in India have documented similar expenditures on tobacco consumption in other populations as well [15–17]. Tobacco cessation activities would arrest the initiation and progression of OPMD into oral cancer, and would eventually allay current and future healthcare costs.

The present study found that rural residence, duration since diagnosis of OPMD, and having the first visit at a private practitioner increased the costs of OPMD. Patients staying in rural areas have to travel to the tertiary care centres, which are usually found in towns or cities, for the care of their OPMD complaints. Also, due to the long travel, there is a possibility of delayed care-seeking and a longer duration of diagnosis since the onset of symptoms. Also, the poor accessibility to specialists in rural areas makes patients seek care at local traditional healers or quacks or private practitioners, thereby adding to the costs incurred for OPMD. These findings were supported by research on the determinants of catastrophic expenditures in patients with cancer [18–20].

We conclude that the costs of OPMD in our setting are low, however, the need for financial protection mechanisms such as universal cash transfers should be explored to overcome the direct non-medical and indirect costs. This is the first study estimating the healthcare costs of patients with OPMD in a sparsely-populated district of western India with a high caseload. Further studies are required in densely-populated urban metropolitan settings of India for estimating costs, and exploring the effects of early management, tobacco cessation, and financial protection on cost mitigation among patients with OPMD.

### Limitations

There are a few inherent limitations of studies estimating costs. Since the costs are estimated based on a survey, recall bias is inherent. However, we interviewed more than 90% of patients within a month of diagnosis to avoid recall bias. We could not use any scale for assessing the severity of OPMD among the patients, which might have differentially affected the costs incurred. Also, costs incurred till the resolution of symptoms or cure or progression could not be included as we did not follow up with the patients. Our study did not employ the ‘cost of illness’ perspective, that is, we did not estimate the financial burden incurred by the hospital for the care of the patients. Since patients taking treatment at a private facility were not included, the study findings can be generalized only to patients being treated at government health facilities in similar semi-urban settings of India.

## Supplementary Information

**Additional file 1.** Table showing the unadjusted linear uni-variable regression analysis for variables predicting total costs of OPMD at Bhavnagar during July-September 2019.

## Data Availability

The datasets generated and/or analysed during the current study are available in the Mendeley repository, https://data.mendeley.com/datasets/kp8tnz6jzz/1.

## Abbreviations

CI: Confidence interval
GATS: Global Adult Tobacco Survey
INR: Indian Rupees
IQR: Interquartile range
OBC: Other backward class
OPD: Outpatient department
OPMD: Oral potentially malignant disorders
OSMF: Oral submucous fibrosis
PI: Principal investigator
SC: Scheduled caste
SLT: Smokeless tobacco
SPSS: Statistical Package for Social Sciences
ST: Scheduled tribe
WHO: World Health Organization

## References

[1] Manne RK (2014). Oral potentially malignant disorders/individuals. Oral Oncol.

[2] Warnakulasuriya S, Johnson NW, Van Der Waal I (2007). Nomenclature and classification of potentially malignant disorders of the oral mucosa. J Oral Pathol Med.

[3] Mello FW, Miguel AFP, Dutra KL, Porporatti AL, Warnakulasuriya S, Guerra ENS (2018). Prevalence of oral potentially malignant disorders: a systematic review and meta-analysis. J Oral Pathol Med.

[4] Pahwa V, Nair S, Shetty RS, Kamath A (2018). Prevalence of oral premalignant lesions and its risk factors among the adult population in Udupi Taluk of coastal Karnataka, India. Asian Pac J Cancer Prev.

[5] Pratik P, Desai VD (2015). Prevalence of habits and oral mucosal lesions in Jaipur, Rajasthan. Indian J Dent Res.

[6] Mehrotra R, Pandya S, Chaudhary AK, Kumar M, Singh M (2008). Prevalence of oral pre-malignant and malignant lesions at a tertiary level hospital in Allahabad, India. Asian Pac J Cancer Prev.

[7] Srivastava R, Sharma L, Pradhan D, Jyoti B, Singh O (2020). Prevalence of oral premalignant lesions and conditions among the population of Kanpur City, India: a cross-sectional study. J Fam Med Prim Care.

[8] Ni Riordain R, Christou J, Pinder D, Squires V, Hodgson T (2016). Cost of illness of oral lichen planus in a UK population—a pilot study. J Oral Pathol Med.

[9] Thomson PJ (2017). Potentially malignant disorders-the case for intervention. J Oral Pathol Med.

[10] Aguilar RR, Antonio J, Saucedo M, Martínez ST (2018). Financial risk of increasing the follow—up period of breast cancer treatment currently covered by the Social Protection System in Health in México. Cost Eff Resour Alloc..

[11] Ventura-Alfaro CE, Torres-Mejía G, Ávila-Burgos LD (2016). Hospitalization and mortality in Mexico due to breast cancer since its inclusion in the catastrophic expenditures scheme. Salud Publica Mex.

[12] Registrar General of India. Ministry of Home Affairs. Government of India. Census of India 2011—district census handbook Bhavnagar: primary census abstract. New Delhi: Government of India; 2011. https://censusindia.gov.in/2011census/dchb/2414_PART_B_DCHB_BHAVNAGAR.pdf.

[13] Hulley S, Cummings S, Browner W, Grady D, Newman T (2013). Designing clinical research: an epidemiologic approach.

[14] World Health Organization (2017). Tuberculosis patient cost surveys: a handbook (Annex 1: generic survey instrument).

[15] Tata Institute of Social Science (TISS) Mumbai and Ministry of Health and Family Welfare. Government of India. Global Adult Tobacco Survey GATS 2. Mumbai; 2017.

[16] Rupani MP, Parikh KD, Kakadia MJ, Pathak MM, Patel MR, Shah MA (2019). Cross-sectional study on smokeless tobacco use, awareness and expenditure in an urban slum of Bhavnagar, western India. Natl Med J India.

[17] Schensul JJ, Nair S, Bilgi S, Cromley E, Kadam V, Mello SD (2013). Availability, accessibility and promotion of smokeless tobacco in a low-income area of Mumbai. Tob Control.

[18] Doshmangir L, Yousefi M, Hasanpoor E, Eshtiagh B, Haghparast-Bidgoli H (2020). Determinants of catastrophic health expenditures in Iran: a systematic review and meta-analysis. Cost Eff Resour Alloc.

[19] Kimman M, Jan S, Yip CH, Thabrany H, Peters SA, Bhoo-Pathy N (2015). Catastrophic health expenditure and 12-month mortality associated with cancer in Southeast Asia: results from a longitudinal study in eight countries. BMC Med.

[20] Leng A, Jing J, Nicholas S, Wang J (2019). Catastrophic health expenditure of cancer patients at the end-of-life: a retrospective observational study in China. BMC Palliat Care.

