# Supplementary material for: Costs incurred by patients with oral potentially malignant disorders: is there a public health need for financial protection in India?
Source: BMC Res Notes. 2021 Oct 24;14:396. doi: 10.1186/s13104-021-05814-2 (PMC8543918; doi:10.1186/s13104-021-05814-2)
Supplement: Supplementary file 1 — Additional file 1. Table showing the unadjusted linear uni-variable regression analysis for variables predicting total costs of OPMD at Bhavnagar during July-September 2019. [file 13104_2021_5814_MOESM1_ESM.docx]

Table: Unadjusted linear uni-variable regression analysis for variables predicting total costs of OPMD at Bhavnagar during July-September 2019 (n=219)

| **Variables** | **Beta-coefficients** | **95% C.I. of beta-coefficients** | **P-value** |
| --- | --- | --- | --- |
| Age in years | 1.011 | 1.004 – 1.019 | 0.002 |
| Male gender | 1.169 | -1.046 – 1.429 | 0.127 |
| Rural residence | 1.272 | 1.078 – 1.502 | 0.005 |
| Years of education | -1.007 | -1.025 – 1.009 | 0.383 |
| Occupation requiring labour | -1.009 | -1.214 – 1.192 | 0.924 |
| Belonging to below poverty line family | -1.146 | -1.359 – 1.034 | 0.117 |
| Sole earner in family | -1.188 | -1.434 – 1.016 | 0.073 |
| OSMF (vs. other OPMD) | 1.059 | -1.152 – 1.292 | 0.571 |
| Number of months since diagnosis | 1.192 | 1.128 – 1.259 | <0.001 |
| Number of years since consuming smokeless tobacco | 1.018 | 1.007 – 1.03 | 0.001 |
| First visit at a private provider | 2.591 | 1.961 – 3.427 | <0.001 |

^OSMF = Oral Submucous Fibrosis; OPMD = Oral Potentially Malignant Disorders^
